# Dominance Effects of Deleterious and Beneficial Mutations in a Single Gene of the RNA Virus ϕ6

**DOI:** 10.1371/journal.pone.0097717

**Published:** 2014-06-19

**Authors:** Sarah B. Joseph, Kayla M. Peck, Christina L. Burch

**Affiliations:** Department of Biology, University of North Carolina, Chapel Hill, North Carolina, United States of America; Centers for Disease Control and Prevention, United States of America

## Abstract

Most of our knowledge of dominance stems from studies of deleterious mutations. From these studies we know that most deleterious mutations are recessive, and that this recessivity arises from a hyperbolic relationship between protein function (i.e., protein concentration or activity) and fitness. Here we investigate whether this knowledge can be used to make predictions about the dominance of beneficial and deleterious mutations in a single gene. We employed a model system – the bacteriophage φ6 – that allowed us to generate a collection of mutations in haploid conditions so that it was not biased toward either dominant beneficial or recessive deleterious mutations. Screening for the ability to infect a bacterial host that does not permit infection by the wildtype φ6, we generated a collection of mutations in P3, a gene involved in attachment to the host and in phage particle assembly. The resulting collection contained mutations with both deleterious and beneficial effects on fitness. The deleterious mutations in our collection had additive effects on fitness and the beneficial mutations were recessive. Neither of these observations were predicted from previous studies of dominance. This pattern is not consistent with the hyperbolic (diminishing returns) relationship between protein function and fitness that is characteristic of enzymatic genes, but could have resulted from a curve of increasing returns.

## Introduction

**Figure 1:**
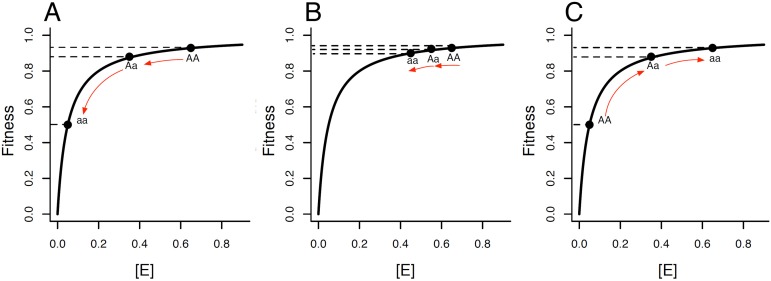
The hyperbolic relationship between enzyme concentration ([*E*]) and fitness is predicted to determine the dominance of mutations affecting enzyme concentration. When fitness of the wildtype is near the plateau of the hyperbolic curve, (A) mutations that substantially reduce enzyme concentration are predicted to be recessive and (B) mutations that slightly reduce enzyme concentration have additive effects. (C) When fitness of the wildtype is lower, it is possible to accumulate mutations that substantially increase enzyme concentration. These mutations are predicted to be dominant over the wildtype allele. These predictions are for mutations that alter enzyme concentration, but can be extended to include mutations that alter other components of protein function, namely protein activity.

Nearly 150 years after Mendel first observed recessive traits in pea plants [1], empirical studies have shown that most deleterious mutations are recessive [2], [3], [4]. The most widely accepted theory for why mutations should be recessive is the Physiological Theory [5], [6], which argues that dominance is a natural result of the physiological mechanics of protein function. For mutations in enzymatic genes, the dominance of the wildtype over most deleterious mutations results, simply, from the hyperbolic relationship between enzyme concentration and flux through a metabolic pathway (see Figure 1). Empirical investigations of mutational effects in enzymes have confirmed that enzyme concentration is hyperbolically related to flux [5], and also to fitness [7]. If we consider the Physiological Theory more generally, the exceptions seem to prove the rule. In cases like Huntington’s disease, where deleterious mutations are dominant, they typically occur in non-enzymatic genes (reviewed in [4]).

While many studies have examined the dominance of deleterious mutations, the rarity of beneficial mutations makes it difficult to perform analogous studies on them without inadvertently selecting for dominant mutations (Haldane's sieve; [8], [9]). In light of these limitations, it is worth considering whether studies of deleterious mutations can inform our knowledge of beneficial mutations. In the specific example described above, if the recessivity of most deleterious mutations is explained by the hyperbolic (diminishing returns) relationship between protein concentration and function that characterizes enzymatic genes, does that mean that most beneficial mutations are also governed by that hyperbolic relationship, causing their effects to be dominant (see Figure 1)? More generally, if the dominance effects of deleterious mutations in a particular gene were known – whether recessive, additive, or dominant – could that knowledge be used to predict the dominance effects of beneficial mutations in the same gene?

In this study, we examine the dominance and selection coefficients of a collection of spontaneous mutations in the bacteriophage φ6. Our collection differs from those of earlier studies in several important ways – the mutations occur primarily in a single gene, span a wide range of fitness effects, and include an unbiased sample of deleterious and beneficial mutations. Thus, we are able to test whether deleterious mutations in this gene are recessive, and beneficial mutations are dominant, as would be predicted by a hyperbolic relationship between protein function and fitness (Figures 1A and 1C).

## Materials and Methods

### Ancestor Strain, Culture Conditions and Archiving

In this study we used two laboratory strains of the double-stranded RNA bacteriophage φ6, both descended from the original isolate [10]. The first strain, φ6_mindich_, was reconstructed from cloned genome segments [11]. The bacteria and plasmids used to construct this strain were supplied by Leonard Mindich (Public Health Research Institute of New Jersey Medical School). The second strain, φ6_37F-41_, was obtained from Lin Chao (University of California, San Diego). φ6_37F-41_ has served as the ancestor for previous evolution experiments [12], [13], [14], [15] and was used here because it generates a wide array of host range mutations during the 5 generations that elapse during formation of single plaques [15]. φ6_37F-41_ has a higher fitness than φ6_mindich_, probably because of differences in laboratory passage. We employed two host bacteria, the standard laboratory host *Pseudomonas syringae* pathovar phaseolicola strain HB10Y, obtained from the American Type Culture Collection (ATCC no. 21781), and a novel host *Pseudomonas syringae* pathovar glycinea strain R4a, obtained from Jeff Dangl (University of North Carolina).

Bacteriophage and their hosts were cultured and titered in standard LC media (5 g yeast extract, 5 g NaCl, and 10 g Bacto-tryptone per liter H_2_O) [13]. Phage were grown on plates by overlaying a mixture of phage, 200 µL of an overnight culture of bacteria, and 3.5 mL top agar (LC+0.7% agar) onto solid media (LC+1.5% agar). Bacteriophage and bacteria were incubated for growth at 25°C, and archived in 40% glycerol at −20°C and −80°C, respectively.

### Host Range Mutants

We isolated host range mutants capable of growth on *Pseudomonas syringae* pathovar glycinea, an alternative host that φ6_mindich_ and φ6_37F-41_ cannot infect. The ancestor phage was plated on the standard laboratory host *P. phaseolicola* to obtain isolated plaques. Phage were harvested from randomly chosen isolated plaques and plated on the alternative bacterial host *P. glycinea*. After 24 hours of growth, a single mutant plaque was frozen per plate. This procedure was repeated to obtain independent mutants. Mutants were then plaque purified by streaking each frozen plaque onto a lawn of the alternative host. One purified plaque per plate was archived by freezing. Mutants of φ6_37F-41_ were isolated in a previous study [15].

We examined host range mutants to identify those with both a different growth rate than their ancestor on the standard laboratory host *P. phaseolicola* and a unique mutation in the attachment gene P3. Differences in growth rate relative to the ancestral phage were identified by visually inspecting plaque sizes on *P. phaseolicola* as in [12], and mutations were identified by amplifying and sequencing the region encompassing nucleotides 1298–3873 of the medium genome segment. This region encompasses the entire P3 and P13 genes and part of the P6 gene [16]. RNA isolation, amplification and sequencing were performed using the protocol described by Ferris *et al.* [15]. That study examined a larger analogous collection of host range mutations and confirmed that the majority (>75%) possessed only the single mutation identified in P3 and no additional second-site mutations [15].

### Burst Size Assays

Burst assays measured the number of offspring produced from individual host cells infected by two bacteriophage (*i.e.* coinfected cells). These assays were performed by incubating 3×10^8^ exponentially growing standard host cells (*P. phaseolicola*) with 4.5×10^9^ phage of a particular mutant genotype (a) and 4.5×10^9^ ancestral phage (A). Phage titers were calculated using a plaque assay on the standard host. After 20 minutes of incubation, we separated coinfected host cells from viruses that had not yet infected a host by centrifuging the mixture for 5 minutes and pouring off the supernatant. This washing procedure was repeated 3 times, resuspending the pelleted cells in 5 ml of LC after the first two washes and in 1 ml of LC after the final wash. The mixture was then diluted and 20 µl volumes were aliquoted into wells of at least five 96-well plates so that on average, 1 out of 10 wells contained an infected cell. The multiwell plates were then incubated for three hours with shaking to allow bacteriophage to lyse their hosts. Wells containing bacteriophage progeny were identified by spotting 3 µl from each well onto a test plate of LC solid media overlayed with top agar and the standard host. The multiwell plate and test plate were then incubated overnight at 4°C and 25°C, respectively.

**Figure 2:**
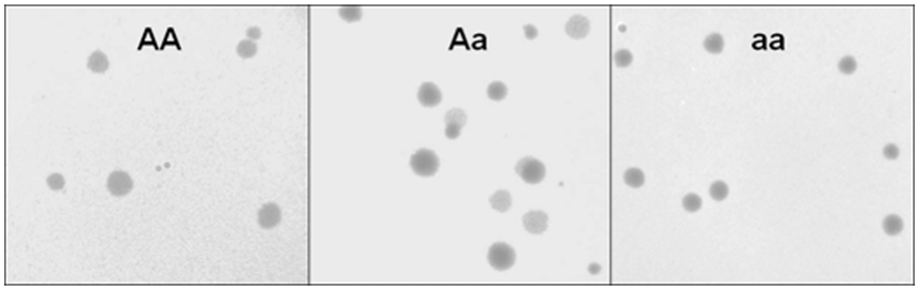
Plaque genotyping assay. Progeny from a single coinfected cell were plated on a mixed lawn of the standard (*P. phaseolicola*) and novel (*P. glycinea*) hosts. Mutant (a) progeny can infect both hosts and result in clear plaques, while wildtype (A) progeny only infect the standard host and result in turbid plaques. The coinfection type can be identified as either AA (only turbid plaques; left panel), Aa (both turbid and clear plaques; center panel) or aa (only clear plaques; right panel).

The following day, the test plate was inspected to identify the wells that contained progeny. We then capitalized on the ability of our mutants to infect the alternative host *P. glycinea* to identify wells that had contained homozygous mutant (aa), heterozygous (Aa) or homozygous wildtype (AA) coinfections. We plated 10 µl (half the total original volume) from each progeny-containing well onto a mixed lawn of the standard (*P. phaseolicola;* 100 µl*)* and alternative *(P. glycinea;* 200 µl) hosts. Mutant (a) progeny form clear plaques on mixed lawns, whereas wildtype (A) progeny form turbid plaques (Figure 2). We incubated these plates overnight at 25°C, characterized the coinfection type as either AA (only turbid plaques), Aa (both turbid and clear plaques), or aa (only clear plaques), and counted the plaques that resulted. Burst size was calculated as twice this total number of plaques. Burst sizes of less than ten were removed from the dataset because the small number of plaques examined from these bursts (<5) made it difficult to accurately categorize the coinfection type. For example, a burst size of eight would correspond to an experimental plate with four plaques. If these progeny were produced by a heterozygous burst that yielded equal numbers of mutant and wildtype offspring then there would be a 0.13 probability of miscategorizing the coinfection type due to sampling only clear or turbid plaques.

### Degree to which Mutations are Complemented by the Wildtype Allele

We define fitness as the number of progeny released from a coinfected cell (*i.e.* burst size). All fitness estimates were standardized by the mean fitness of homozygous wildtype coinfections (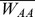) in that assay. This controlled both for the effects of assay-to-assay variation and for fitness differences between the two ancestor genotypes.

We then examined the degree to which each mutation was complemented (*h*) in heterozygous coinfections by comparing the fitness effects of mutations in homozygous mutant coinfections (*s*) to their effects in heterozygous coinfections (*hs*), where
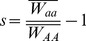
(1)and
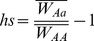
(2)


### Statistical Analyses

Statistical analyses of these data were performed using R statistical software (version 2.6.2). Raw fitness (i.e. burst size) data were corrected for day effects by dividing each individual fitness measure by the mean fitness of all AA coinfections measured on the same day. After correcting for day effects we used the individual *log* (relative mean fitness) values to obtain estimates and standard errors of the quantities *log* 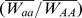 and *log* 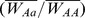. These quantities were then back transformed to obtain means and standard errors of 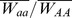 and 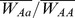 for use in eqs. (1) and (2) to estimate *s* and *hs* for plotting and further analysis.

### Predicted Dominance Relationships

**Figure 3:**
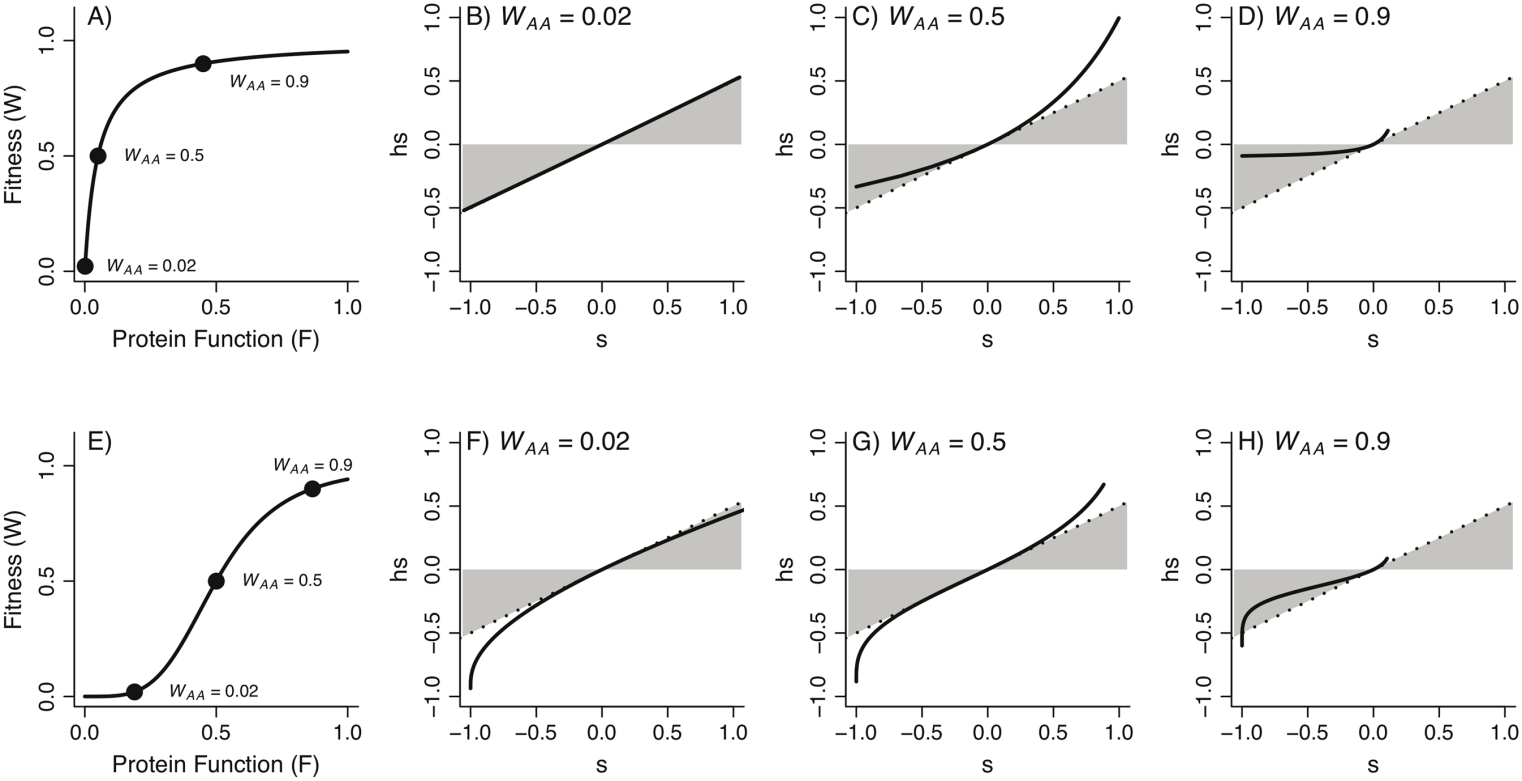
The relationship between protein function and fitness alters the complementation of mutations affecting protein function. Regions where mutations have recessive effects (0< *h*<0.5) are shown in gray and additive effects (*h* = 0.5) are shown as a dashed line. The Physiological Theory predicts that (A) the hyperbolic relationship between protein function and fitness results in additive mutation effects only when the wildtype fitness is near zero (B). At moderate (C) and high (D) wildtype fitnesses, deleterious and beneficial mutations are predicted to be recessive and dominant, respectively. The sigmoidal fitness function predicted for proteins that display cooperative binding (E) causes a stronger dependence of dominance coefficients on wildtype fitness. For instance, the sigmoidal relationship yields recessive beneficial mutations when wildtype fitness is low (F), but dominant beneficial mutations when wildtype fitness is moderate (G) and high (H).

We first ensured that we had a solid understanding of the theoretically predicted dominance relationships by exploring how the biochemical relationship between protein function and fitness translates into the dominance relationship between the homozygous effects (*s*) and heterozygous effects (*hs*) of mutations. We examined two hypothetical relationships between protein function and fitness – one hyperbolic and one sigmoidal – to calculate the expected dominance of mutations affecting protein function. The hyperbolic fitness function was based on the Michaelis-Menten equation from enzyme kinetics
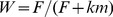
(3)and states that fitness (*W*) is a function of protein function (*F*) and the Michaelis constant (*km*). In Figure 3A we graph this equation for *km* = 0.5. The sigmoidal fitness function was based on the Hill equation that describes cooperative binding
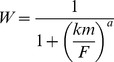
(4)In equation (4) fitness is also determined by the Hill coefficient *a*, which describes cooperativity and determines the steepness of the transition from low to high fitness. Hill coefficients governing virus capsid assembly range from 2 in Sindbis virus [17] to 6 in SV40 [18]. In Figure 3E we graph this equation for an intermediate value of *a* = 4 and *km* = 0.5.

The hyperbolic (eq. 3) and sigmoidal (eq. 4) fitness functions were evaluated at three wildtype fitnesses (*W_AA_* = 0.02, *W_AA_* = 0.5, and *W_AA_* = 0.9) to determine the resulting relationships between the heterozygous (*hs*) and homozygous (*s*) effects of mutations that alter protein function. We assumed that protein function is additive and used equations (3) and (4) to identify the wildtype protein function that yields a particular wildtype fitness (*W_AA_* = 0.02, 0.5, or 0.9) and then examined the degree to which mutations that alter protein function are masked during coinfection.

For example, if *W_AA_* = 0.5, *km* = 0.5 and the relationship between protein function and fitness is hyperbolic, then equation (3) can be rearranged and solved for *F* ( = 0.125 in this example). If a mutation renders this protein nonfunctional, then the heterozygote will have half the protein function of a homozygous wildtype (*F* = 0.063 in this example) and the homozygous mutant will have no protein function (*F* = 0). We then used these values to solve equation (3) and calculate fitness of the heterozygote, *W_Aa_*, and the homozygous mutant, *W_aa_*. Finally, we inferred *hs* and *s* from the three fitness values (*W_AA_*, *W_Aa_* and *W_aa_*). This procedure was repeated for mutations (both deleterious and beneficial) that cause fitness of the homozygous mutant to range from 0 to 1.

The resulting relationship between *hs* and *s* is shown for three wildtype fitness values *W_AA_* = 0.02, 0.5, and 0.9 for both the hyperbolic fitness function (Figure 3 B–D) and the sigmoidal fitness function (Figure 3 F–H).

## Results

### Generating a Collection of Mutations

Our goal was to test the predictions that arise from a hyperbolic fitness function (Figure 3A–D) by examining the dominance of mutations that occur within an individual gene and continuously span a wide range of homozygous fitness effects, from strongly deleterious to strongly beneficial. Simply obtaining such a collection of mutations is challenging in most organisms. To overcome this challenge, we capitalized on the observation that in the bacteriophage φ6, a phenotypic screen for mutations that expand host range yields a large collection of different mutations, mostly in the host attachment gene P3 [15], [19]. We conducted this mutation screen using two phage genotypes, one of intermediate and one of high fitness in lab culture, to ensure that the resulting collection contained mutations with a wide range of effects from very deleterious to very beneficial.

To obtain a collection of mutations in a single gene, we mixed a ‘wildtype’ lab strain (φ6_mindich_) with a host that it is incapable of infecting and isolated 25 mutants capable of growth on this alternative host. We then returned the mutant phage to the standard host and examined the plaques that formed. Plaque size has been shown to be an exceptionally good indicator of the log(number of viruses) within a plaque [12]. 19 of our 25 mutants had plaque sizes that differed from the wildtype phage.

We sequenced the attachment gene P3 in these 19 mutants to identify the mutations responsible for host range expansion. Previous studies have shown that most host range mutations in φ6 occur in the P3 gene [15], [20]. Similarly, 17 of the 19 φ6_mindich_ mutants had mutations in P3, and 12 of them were unique (Table 1). We concluded that the two mutants lacking a mutation in P3 are genetically different because they have significantly different effects on homozygous fitness (see below).

**Table 1:** Mutation identity.

Host range mutant	Nucleotide substitution in P3^a^	Amino acid substitution in P3^a^	Ancestor
HR2	–	–	φ6_mindich_
HR3^b^	A125G	K42R	φ6_mindich_
HR4	A1211G	E404G	φ6_mindich_
HR5	A1211C	E404A	φ6_mindich_
HR6	A1709G	D570G	φ6_mindich_
HR8	G1228A	D410N	φ6_mindich_
HR9^c^	T136C	F46L	φ6_mindich_
HR10	G1708A	D570N	φ6_mindich_
HR14^c^	A1229G	D410G	φ6_mindich_
HR15	T136A	F46I	φ6_mindich_
HR16^c^	T137C	F46S	φ6_mindich_
HR19	A536G	D179G	φ6_mindich_
HR23	–	–	φ6_mindich_
HR25	T620C	F207S	φ6_mindich_
G25	A23G	E8G	φ6_37F-41_
G27	C1016T	P339H	φ6_37F-41_
G28	A1661C	D554A	φ6_37F-41_

a Substitutions are labeled relative to their position in P3.

b Two additional host range mutants had the same substitution.

c One additional host range mutant had the same substitution.

We added to this collection three host range mutants from a previous experiment [15] with known deleterious mutations of large effect. These additional mutants were obtained in an identical manner, but were derived from a different ancestor, φ6_37F-41_. They are designated with a “G” before the mutant number (Table 1).

### Homozygous and Heterozygous Effects of Mutations

A second experimental hurdle that arises in investigations of dominance is that dominance coefficients (*h*) are hard to measure. In particular, obtaining independent estimates of selection (*s*) and dominance (*h*) coefficients from measurements of the fitness of three diploid genotypes (*W_AA_*, *W_Aa_*, and *W_aa_*) poses a statistical challenge. The difficulty becomes apparent just by writing down the manner in which *s* and *h* are calculated from fitness measures:
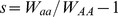


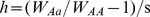
As a result, slight underestimates of *s* can cause large overestimates of *h*.

To overcome this problem, we follow the approach of Szafraniec *et al.* [21] and compare the homozygous (*s*) and heterozygous (*hs*) effects of mutations. One can gain intuition for how the relationship between protein function and fitness translates into the relationship between *s* and *hs*, by assuming a particular relationship between protein function and fitness, and examining the dominance patterns that result. We consider the consequences of hyperbolic and sigmoidal fitness functions for the dominance of mutations arising in wildtype (AA) individuals with three different fitness values (Figure 3), and illustrate that dominance effects depend critically on both the shape of the fitness function and the fitness of the wildtype.

When the fitness function is hyperbolic (Figure 3A), as has been observed for enzymes [5], [7] deleterious mutations that reduce protein function are recessive, falling in the gray region that corresponds to 0<*h*<0.5 in Figures 2B–2D. In contrast, beneficial mutations that increase protein function are dominant, falling in the region that corresponds to *h*>0.5. In the hyperbolic case, the *quantitative* relationship between *hs* and *s* depends on the fitness of the wildtype. Both recessive and beneficial mutations become more additive as wildtype fitness declines toward zero. When the relationship is not hyperbolic, other patterns are possible. For instance, if the relationship between protein function and fitness is sigmoidal (Figure 3E), as may occur in cooperatively-binding allosteric proteins, then beneficial and deleterious mutations can be either dominant or recessive, and the *qualitative* relationship between *hs* and *s* depends on the fitness of the wildtype (Figures 2F–2H).

### Coinfecting Hosts

We examined whether mutations were complemented during coinfections by comparing the effects of mutations when hosts were coinfected by two mutant bacteriophage (aa) and when hosts were coinfected by a wildtype and a mutant phage (Aa). Specifically, we measured the effects of mutations on burst size, the number of progeny viruses released by an infected cell – a major component of fitness in viruses. Coinfections were initiated by mixing mutant (a) and wildtype (A) phage with host cells at a high multiplicity of infection (MOI, the ratio of bacteriophage to hosts). The high MOI ensured that all cells were coinfected very rapidly. Furthermore, in φ6, coinfection is limited to two phage per cell [22], [23] – making coinfection similar to diploidy. After allowing sufficient time for phage to infect cells, this mixture was divided into aliquots so that, on average, only one out of every 10 aliquots contained an infected cell. Infected cells were incubated until lysis, and then each aliquot was plated onto a bacterial lawn that allowed us to count the number of progeny released (*i.e*. the burst size), and to determine whether the progeny phage were of a single genotype, or whether they were a mixture of wildtype (A) and mutant (a) phage.

We compared the coinfection type frequencies (AA, Aa and aa) that emerged from each coinfection assay to the Hardy-Weinberg expectation (Table S1) to ensure that cells were independently infected by two phage. Frequencies are expected to deviate from the Hardy-Weinberg expectation if cells were infected by single phage (scored as excess of homozygotes) or by more than two phage (scored as excess of heterozygotes). Data from 4 assays out of 39 total showed a significant (p<0.05) deviation from the Hardy Weinberg expectation, but this was not unexpected given the large number of comparisons. Using the Bonferroni method to correct for multiple comparisons, none of the assays remain significant at p<0.001. In addition, the observed number of heterozygous coinfections was sometimes greater and other times less than the Hardy-Weinberg expectation, suggesting that there was no systematic bias in the data and that most cells were infected by two phage.

We also confirmed that our experimental design ensured that phage simultaneously infected hosts. This is a concern primarily because mutations in P3, the host attachment gene, are expected to affect the rate of host attachment. In φ6, the genome segments of coinfecting phage are known to package randomly, without bias, into progeny virions [24]. As a result, if cells were infected by two phage simultaneously and those phage replicate at the same rate, then each phage should contribute equally to the resulting progeny. We calculated the frequency of mutant progeny, *f* (a), produced from each heterozygous coinfection (Table 2). For particular mutants, the average *f* (a) ranged from 0.42 to 0.56, with 4 mutants out of 16 showing a small but significant deviation from 0.5. The mean over all mutants, *f* (a)  = 0.48, deviated from the 0.5 expectation but the deviation was small relative to the magnitude of mutational effects (see below).

**Table 2:** Coinfection data.

			Standardized fitness		Mean *f* (*a*)from heterozygous coinfections*^a^*
Mutant	Coinfection type	Observed number ofcoinfected cells	Mean	95% confidence interval	
HR2	aa	8	0.51	(0.36, 0.73)	
"	Aa	32	0.84	(0.68, 1.04)	NA*^b^*
"	AA	25	1	(0.79, 1.26)	
HR3	aa	11	1.2	(0.92, 1.59)	
"	Aa	44	0.94	(0.76, 1.15)	0.45±0.08
"	AA	27	1	(0.79, 1.26)	
HR4	aa	17	0.43	(0.31, 0.60)	
"	Aa	49	0.71	(0.59, 0.86)	0.43±0.04*
"	AA	42	1	(0.87, 1.14)	
HR5	aa	17	0.4	(0.27, 0.58)	
"	Aa	43	0.72	(0.56, 0.92)	0.49±0.08
"	AA	9	1	(0.67, 1.49)	
HR6	aa	22	0.93	(0.73, 1.18)	
"	Aa	31	0.88	(0.73, 1.06)	0.47±0.04
"	AA	22	1	(0.74, 1.34)	
HR8	aa	19	1.17	(0.88, 1.54)	
"	Aa	36	1.03	(0.82, 1.30)	0.48±0.04
"	AA	9	1	(0.62, 1.62)	
HR9	aa	11	0.8	(0.58, 1.11)	
"	Aa	22	0.78	(0.60, 1.00)	0.44±0.14
"	AA	32	1	(0.83, 1.21)	
HR10	aa	40	0.95	(0.79, 1.13)	
"	Aa	48	1.07	(0.91, 1.26)	0.44±0.04*
"	AA	38	1	(0.86, 1.17)	
HR14	aa	28	1.37	(1.12, 1.68)	
"	Aa	53	1	(0.82, 1.23)	0.46±0.07
"	AA	54	1	(0.85, 1.18)	
HR15	aa	28	0.66	(0.48, 0.89)	
"	Aa	38	1.03	(0.84, 1.26)	0.46±0.04*
"	AA	29	1	(0.84, 1.19)	
HR16	aa	43	1.15	(0.90, 1.46)	
"	Aa	94	0.89	(0.77, 1.03)	0.52±0.06
"	AA	43	1	(0.83, 1.20)	
HR19	aa	14	1.41	(1.04, 1.92)	
"	Aa	50	1.02	(0.86, 1.22)	0.47±0.09
"	AA	20	1	(0.78, 1.28)	
HR23	aa	34	0.8	(0.64, 1.01)	
"	Aa	53	0.88	(0.70, 1.09)	0.56±0.07
"	AA	27	1	(0.75, 1.34)	
HR25	aa	13	0.83	(0.64, 1.08)	
"	Aa	24	1.05	(0.85, 1.30)	0.56±0.12
"	AA	16	1	(0.75, 1.33)	
G25	aa	22	0.39	(0.29, 0.51)	
"	Aa	44	0.69	(0.60, 0.79)	0.49±0.05
"	AA	17	1	(0.87, 1.15)	
G27	aa	17	0.73	(0.53, 1.01)	
"	Aa	34	0.84	(0.68, 1.03)	0.47±0.07
"	AA	9	1	(0.54, 1.86)	
G28	aa	10	0.56	(0.27, 1.13)	
"	Aa	36	0.88	(0.72, 1.07)	0.42±0.06*
"	AA	12	1	(0.78, 1.29)	

*a* – Mean and SEM values calculated from the frequency of mutants produced by individual heterozygous coinfections; *indicate intervals that do not include 0.50.

*b –*Not available.

The burst sizes of homozygous mutant (aa), heterozygous (Aa) and homozygous wildtype (AA) coinfections confirmed that there were both deleterious and beneficial mutations in our collection. The mean homozygous effects (*s*) of the mutants ranged from −0.61 to 0.41 and their mean heterozygous effects (*hs*) ranged from −0.31 to 0.07 (Table 2). The smaller range of fitness effects in heterozygous coinfections suggests that the majority of mutations are either additive or recessive.

**Figure 4:**
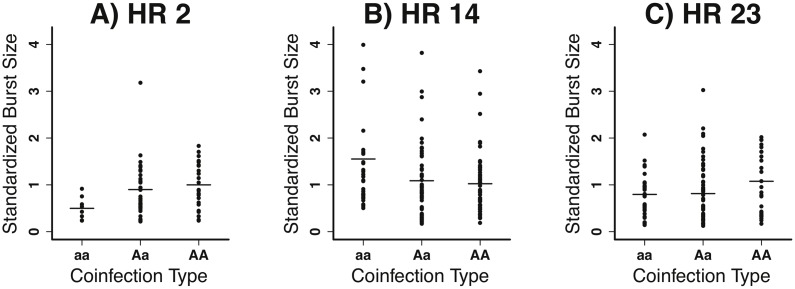
Burst size measures for homozygous mutant (aa), heterozygous (Aa) and homozygous wildtype (AA) coinfections are shown as columns of points and their means are show as lines. Two of the host range mutants (HR2 and HR23) did not have mutations in P3, but differences in their mean homozygous effects suggest that they are different mutations.

To give a better sense of the nature of the data, the entire collection of burst size measurements for three of the mutants is shown in Figure 4. Among other things, these graphs illustrate that burst sizes are highly variable. Such variation cannot be explained by some aliquots containing more than one host cell. Aliquots containing two cells would have a 75% chance of being scored as heterozygous, thus substantially increasing the variance of heterozygous coinfections. The fact that heterozygous and homozygous coinfections have approximately equal variances in our study suggests that the aliquots rarely contained multiple cells. This variation means that we cannot be confident that we have accurately estimated the fitness (and dominance) effects of any individual mutation. We avoid this pitfall by inferring information about dominance from the fitness effects of multiple mutations (see below).

Two of the mutations (HR2 and HR23) were not in P3, but the significant difference between their homozygous effects (Welch’s *t*_19.64_ = −2.30, *p* = 0.03) strongly suggests that they are different mutations.

### Complementing Mutations during Coinfection

**Figure 5:**
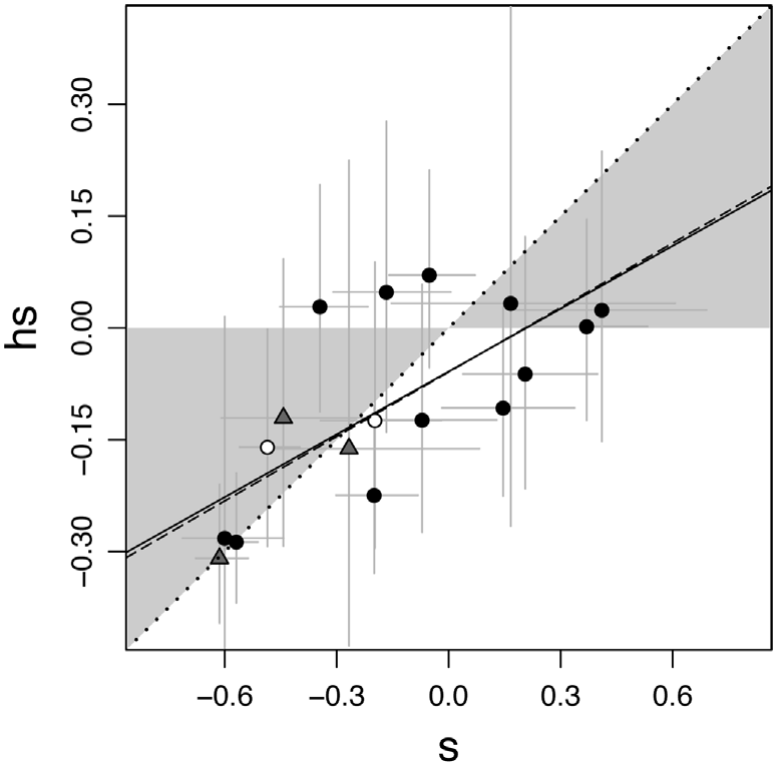
Fitness effects of mutations in heterozygous (*hs*) and homozygous (*s*) coinfections. Data are means ± standard errors of the mean. Regions where mutations have recessive effects (0< *h*<0.5) are shown in gray and additive effects (*h* = 0.5) are shown as a dotted line. The solid line is the reduced major-axis regression line and falls primarily in the recessive region. Mutations are represented as circles if they were accumulated in the φ6_mindich_ background and as triangles if they were accumulated in φ6_37-F41_. Removal of the two mutants that did not have mutations in P3 (white-filled circles) barely affected the major-axis regression (dashed line). Effects of P3 mutations that were obtained and measured in an alternative, higher fitness genetic background are shown with dark gray circles.

In order to examine whether most of our mutations are recessive, we compared the mean effects of individual mutations in homozygous coinfections (*s*) to their mean effects in heterozygous coinfections (*hs*) (Figure 5). The most appropriate way to analyze the relationship between *s* and *hs* is to use a reduced major-axis regression, because both *s* and *hs* were measured with experimental error [25]. The best fit line, or reduced major-axis, is statistically equivalent to the first principle-component axis. In our dataset, the reduced major-axis falls almost entirely within the recessive region of the graph (solid line in Figure 5; recessive region shown in gray). The location of the reduced major-axis was similar when we removed the two mutants that did not have mutations in P3 (dashed line in Figure 5).

**Figure 6:**
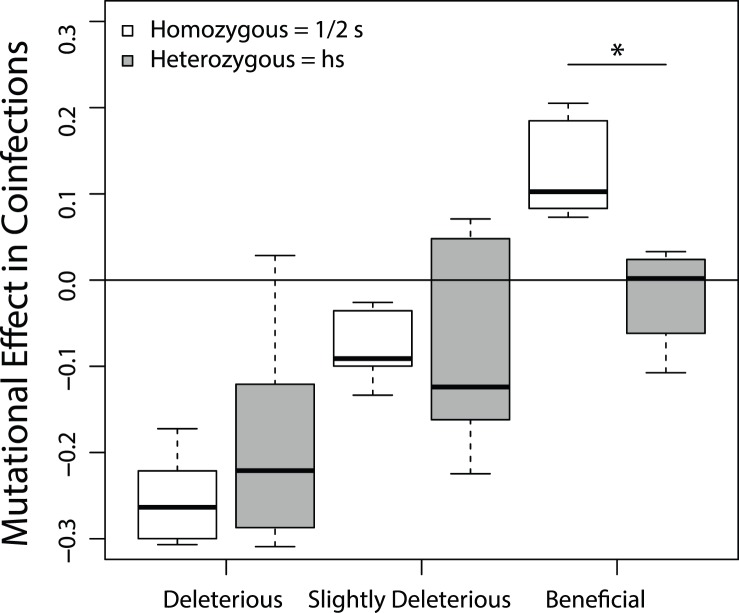
Deviations from additivity. Phage were divided into three bins based on their fitness effects in homozygous coinfections: deleterious (*s*<−0.3, *n* = 5), slightly deleterious (0.3<*s*<0, *n* = 6), or beneficial (*s*>0, *n* = 5). For mutations in each bin, boxplots show either the marginal effects of mutations in homozygous coinfections (½ s) or the fitness effects of mutations in heterozygous coinfections (*hs*). *p*-values resulted from paired Welch’s *t*-tests that tested the additive expectation that these two quantities were equal (i.e. *hs* = ½ *s*). * Indicates the only statistically significant comparison between homozygous and heterozygous effects of mutations (*p* = 0.0041).

Although the reduced major-axis falls in the recessive region of the graph for both deleterious and beneficial mutations, a qualitative assessment of the individual data points by eye suggested that this pattern might be driven primarily by the beneficial mutations. We assessed this possibility by dividing the data into three bins - deleterious (s<−0.3), slightly deleterious (−0.3<s<0), and beneficial (s>0) – and testing for a significant deviation from additivity using a paired Welch’s *t*-test to compare 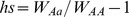 to the additive expectation 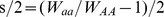 for each mutation within each bin (Figure 6). Although the effects of deleterious and slightly deleterious mutations were not statistically distinguishable from additivity (*p*>0.05), the effects of beneficial mutations were significantly smaller in heterozygous coinfections than the additive expectation (*p* = 0.0041) – i.e. beneficial mutations are recessive.

## Discussion

In this study, we measured the dominance coefficients of a collection of mutations obtained by screening for effects on a single phenotype (host range). The mutations occurred primarily in the host attachment gene P3. We observe that on average, deleterious mutations in P3 act additively whereas beneficial mutations in P3 are recessive. If these mutations alter the same component of P3 protein function, then their dominance should stem from a single curve relating that component to fitness. The hyperbolic curve that has often been used to explain the dominance patterns of deleterious mutations is consistent with additive deleterious mutations if the wildtype phage has a low fitness, but there is no scenario in which a hyperbolic curve can produce recessive beneficial mutations (See Figures 2A–2D).

Although the phage model system is experimentally tractable, it makes an unconventional choice for examining dominance, which typically only affects diploid organisms. Although phage are not diploid organisms, when two phage coinfect a host cell it creates a condition that is mechanistically analogous to diploidy, in that offspring are produced from a common resource pool composed of proteins translated from two homologous alleles. It has long been recognized that when two phage infect the same host cell, an allele carried by one phage may complement, or mask, the phenotypic effects of the homologous allele carried by the coinfecting phage [26]; a phenomenon that is mechanistically analogous to dominance. The potential for complementation exists for most φ6 phenotypes, but not all. Mutations that affect only attachment to the host cannot be complemented [19] because the virus is haploid during that part of the life cycle, whereas mutations that affect intracellular functions from replication to phage assembly to host lysis can be complemented [27]. Note that our collection of mutations is in the host attachment protein P3, and that any complementation we observe must result from the involvement of P3 in parts of the life cycle (like phage assembly; [28]) that occur downstream of attachment to the host.

Our observation of recessive beneficial mutations is unlikely to have arisen from differences between coinfection and diploidy. In particular, we confirmed that our experimental design ensured that hosts were infected by exactly two phage, and that these two phage infected the host almost simultaneously. As a result, there should not have been differences in the timing of expression of the two homologous alleles that made it look like the beneficial mutations were not being expressed.

Although the nature of the evidence is often anecdotal, the number of anecdotes that support the Physiological Theory, *i.e*. the connection between dominance effects and specific protein function, is growing (reviewed by [4], [29] but see [30]). Our data provide an additional anecdote. Our observation of additive deleterious and recessive beneficial mutations within a single gene is consistent with a curve of increasing returns (*e.g.* an exponential curve) relating protein function to fitness. Although this pattern is not characteristic of enzymes [5], [7] the pattern must have an underlying physiological basis. Studies of the quasi-catalytic functions of non-enzymatic proteins have revealed that a variety of relationships between protein concentration and flux are possible [31], [32], [33]. Of particular interest are sigmoid patterns (Figure 3E) which can emerge from stochastically expressed developmental genes [34], autoregulatory expression networks [31] or cooperative substrate binding [35]. Sigmoid curves should generate different patterns of dominance depending on the fitness of the wildtype and the effect size of a mutation (Figures 2F–2H), and could yield the pattern of additive deleterious and recessive beneficial mutations that we observed.

Our knowledge of the functional roles of the host attachment gene P3 is consistent with a sigmoidal curve resulting from cooperative binding. P3 is a multimeric structural protein that is an integral part of the phage particle, and known to play at least two functional roles: attachment to the host receptor and phage assembly [28]. Our burst size assay did not capture parts of the phage life cycle that occur outside the cell, so it would not have been sensitive to differences in attachment to the host. Rather, it should have captured only mutational effects on intracellular parts of the phage life cycle, like phage assembly.

Mutations that affect assembly are known to have severe fitness consequences in better-studied viruses like HIV [36], [37], [38], [39], [40], and are expected to have similar effects in φ6. Like P3, the HIV capsid proteins assemble into a multimeric structure (reviewed by [41]). Integrity of these multimeric structures requires that capsid proteins interact in specific ways and mutations that alter these interactions typically have large fitness effects [38]. These are the fitness effects captured by our burst size assay.

Although the kinetics of P3’s role in phage assembly are not known, other aspects of phage assembly in φ6 are known to be cooperative [42]. Future experiments could test the hypothesis that emerges from our data – that dominance patterns in P3 are governed by a sigmoidal curve – by redoing our experiment starting with a higher fitness phage. If this hypothesis is correct, we expect the data to converge on the hyperbolic prediction as fitness increases (see Figure 3).

Although our data are not inconsistent with Wright’s idea that the dominance of wildtype alleles over mutant alleles is due to the underlying physiology of gene action [5], [6], they confirm that the physiological properties underlying dominance may be complex and specific to the function being altered and the magnitude of the fitness effect. It is notable that the strongest inconsistency in our data from the hyperbolic expectation comes from the dominance properties of beneficial mutations. Note that the inclusion of beneficial mutations in our collection was possible only because we initiated the study with a relatively low fitness wildtype (unmutated) phage. Thus, our observation of recessive beneficial mutations highlights two important weaknesses of most previous studies. Most examined mutations with only a small subset of selection coefficients - generally either lethal or slightly deleterious mutations [2], [43] and none have examined the impact of variation in wildtype fitness. More work is needed to determine whether our observations are unique to the gene and the system that we examined, or whether dominance patterns often deviate from the hyperbolic curves common to enzymes.

## Supporting Information

Table S1 Hardy-Weinberg analyses of coinfection type frequencies.
(DOC)
