# Supplementary material for: Dominance Effects of Deleterious and Beneficial Mutations in a Single Gene of the RNA Virus ϕ6
Source: PLoS One. 2014 Jun 19;9(6):e97717. doi: 10.1371/journal.pone.0097717 (PMC4063744; doi:10.1371/journal.pone.0097717)
Supplement: Table S1 — Hardy-Weinberg analyses of coinfection type frequencies. (DOC) [file pone.0097717.s001.doc]

**Table S1. Hardy*–*Weinberg test to determine whether cells are independently infected by two phage**

| **Mutant** | **Trial number** | **Coinfection type** | **Observed number of coinfected cells** | **Hardy-Weinberg** | | **Standardized fitness** | | **Mean *f(a)* from hetero-zygous coinfections*^a^*** |
| --- | --- | --- | --- | --- | --- | --- | --- | --- |
|  |  |  |  | **Chi square value** | ***p* value** | **Mean** | **95% confidence interval** |  |
| HR2 | 1 | aa | 2 | 0.09 | 0.76 | 0.50 | (0.40, 0.63) | NA*^b^* |
|  |  | Aa | 4 |  |  | 0.88 | (0.54, 1.45) |  |
|  |  | AA | 3 |  |  | 1.00 | (0.67, 1.48) |  |
|  | 2 | aa | 3 | 0.69 | 0.40 | 0.60 | (0.28, 1.29) | NA*^b^* |
|  |  | Aa | 14 |  |  | 0.88 | (0.62, 1.25) |  |
|  |  | AA | 8 |  |  | 1.00 | (0.63, 1.59) |  |
|  | 3 | aa | 3 | 0.03 | 0.85 | 0.45 | (0.23, 0.89) | NA*^b^* |
|  |  | Aa | 14 |  |  | 0.79 | (0.56, 1.10) |  |
|  |  | AA | 14 |  |  | 1.00 | (0.72, 1.39) |  |
| HR3 | 1 | aa | 6 | 2.70 | 0.10 | 1.07 | (0.74, 1.54) | 0.54 ± 0.10 |
|  |  | Aa | 24 |  |  | 0.77 | (0.55, 1.07) |  |
|  |  | AA | 8 |  |  | 1.00 | (0.79, 1.26) |  |
|  | 2 | aa | 5 | 0.01 | 0.94 | 1.39 | (0.91, 2.13) | 0.34 ± 0.14* |
|  |  | Aa | 20 |  |  | 1.19 | (1.00, 1.42) |  |
|  |  | AA | 19 |  |  | 1.00 | (0.73, 1.38) |  |
| HR4 | 1 | aa | 4 | 0.03 | 0.86 | 0.39 | (0.20, 0.76) | 0.50 ± 0.05 |
|  |  | Aa | 9 |  |  | 0.40 | (0.24, 0.66) |  |
|  |  | AA | 6 |  |  | 1.00 | (0.75, 1.33) |  |
|  | 2 | aa | 3 | 0.78 | 0.38 | 0.55 | (0.42, 0.72) | 0.45 ± 0.08 |
|  |  | Aa | 20 |  |  | 0.92 | (0.75, 1.12) |  |
|  |  | AA | 17 |  |  | 1.00 | (0.84, 1.19) |  |
|  | 3 | aa | 0 | 0.31 | 0.58 | NA*^b^* | NA*^b^* | 0.39 ± 0.12 |
|  |  | Aa | 3 |  |  | 1.44 | (0.88, 2.35) |  |
|  |  | AA | 7 |  |  | 1.00 | (1.53, 1.44) |  |
|  | 4 | aa | 10 | 0.62 | 0.43 | 0.42 | (0.25, 0.69) | 0.40 ± 0.05* |
|  |  | Aa | 17 |  |  | 0.64 | (0.48, 0.84) |  |
|  |  | AA | 12 |  |  | 1.00 | (0.73, 1.36) |  |
| HR5 | 1 | aa | 8 | 0.26 | 0.61 | 0.32 | (0.17, 0.62) | 0.68 ± 0.11* |
|  |  | Aa | 9 |  |  | 0.32 | (0.22, 0.47) |  |
|  |  | AA | 4 |  |  | 1.00 | (0.73, 1.36) |  |
|  | 2 | aa | 9 | 8.73 | 0.003 | 0.49 | (0.33, 0.72) | 0.44 ± 0.10 |
|  |  | Aa | 34 |  |  | 0.89 | (0.68, 1.15) |  |
|  |  | AA | 5 |  |  | 1.00 | (0.49, 2.05) |  |
| HR6 | 1 | aa | 3 | 0.04 | 0.85 | 0.89 | (0.43, 1.85) | 0.56 ± 0.07 |
|  |  | Aa | 7 |  |  | 0.92 | (0.64, 1.32) |  |
|  |  | AA | 5 |  |  | 1.00 | (0.31, 3.23) |  |
|  | 2 | aa | 5 | 0.001 | 0.97 | 1.39 | (0.94, 2.05) | 0.42 ± 0.04* |
|  |  | Aa | 12 |  |  | 0.76 | (0.50, 1.16) |  |
|  |  | AA | 7 |  |  | 1.00 | (0.71, 1.42) |  |
|  | 3 | aa | 14 | 3.8 | 0.05 | 0.82 | (0.60, 1.10) | 0.48 ± 0.06 |
|  |  | Aa | 12 |  |  | 0.98 | (0.86, 1.12) |  |
|  |  | AA | 10 |  |  | 1.00 | (0.75, 1.34) |  |
| HR8 | 1 | aa | 14 | 0.001 | 0.97 | 1.22 | (0.86, 1.72) | 0.40 ± 0.12 |
|  |  | Aa | 20 |  |  | 1.31 | (1.00, 1.71) |  |
|  |  | AA | 7 |  |  | 1.00 | (0.54, 1.86) |  |
|  | 2 | aa | 5 | 3.97 | 0.04 | 1.03 | (0.66, 1.62) | 0.59 ± 0.13 |
|  |  | Aa | 16 |  |  | 0.77 | (0.54, 1.10) |  |
|  |  | AA | 2 |  |  | 1.00 | (0.61, 1.64) |  |
| HR9 | 1 | aa | 8 | 5.00 | 0.02 | 0.67 | (0.48, 0.93) | 0.52 ± 0.18 |
|  |  | Aa | 12 |  |  | 0.76 | (0.55, 1.05) |  |
|  |  | AA | 21 |  |  | 1.00 | (0.82, 1.22) |  |
|  | 2 | aa | 3 | 0.09 | 0.76 | 1.29 | (0.71, 2.34) | 0.34 ± 0.20 |
|  |  | Aa | 10 |  |  | 0.79 | (0.51, 1.22) |  |
|  |  | AA | 11 |  |  | 1.00 | (0.66, 1.51) |  |
| HR10 | 1 | aa | 5 | 0.24 | 0.63 | 0.90 | (0.57, 1.41) | 0.52 ± 0.10 |
|  |  | Aa | 7 |  |  | 0.93 | (0.54, 1.58) |  |
|  |  | AA | 4 |  |  | 1.00 | (0.66, 1.52) |  |
|  | 2 | aa | 8 | 3.52 | 0.06 | 1.04 | (0.77, 1.40) | 0.44 ± 0.07 |
|  |  | Aa | 7 |  |  | 1.44 | (1.16, 1.79) |  |
|  |  | AA | 8 |  |  | 1.00 | (0.89, 1.13) |  |
|  | 3 | aa | 10 | 1.69 | 0.19 | 1.21 | (0.82, 1.77) | 0.36 ± 0.11* |
|  |  | Aa | 10 |  |  | 1.10 | (0.56, 1.44) |  |
|  |  | AA | 7 |  |  | 1.00 | (0.50, 2.01) |  |
|  | 4 | aa | 17 | 2.38 | 0.12 | 0.80 | (0.60, 1.06) | 0.44 ± 0.04* |
|  |  | Aa | 24 |  |  | 1.10 | (0.90, 1.36) |  |
|  |  | AA | 19 |  |  | 1.00 | (0.84, 1.19) |  |
| HR14 | 1 | aa | 11 | 0.91 | 0.34 | 1.70 | (1.16, 2.50) | 0.35 ± 0.10* |
|  |  | Aa | 20 |  |  | 0.87 | (0.57, 1.32) |  |
|  |  | AA | 16 |  |  | 1.00 | (0.73, 1.37) |  |
|  | 2 | aa | 17 | 3.69 | 0.05 | 1.19 | (0.97, 1.46) | 0.53 ± 0.10 |
|  |  | Aa | 33 |  |  | 1.09 | (0.89, 1.35) |  |
|  |  | AA | 38 |  |  | 1.00 | (0.82, 1.22) |  |
| HR15 | 1 | aa | 9 | 0.12 | 0.73 | 0.33 | (0.20, 0.54) | 0.48 ± 0.04 |
|  |  | Aa | 14 |  |  | 0.75 | (0.49, 1.13) |  |
|  |  | AA | 7 |  |  | 1.00 | (0.69, 1.44) |  |
|  | 2 | aa | 6 | 0.87 | 0.35 | 0.42 | (0.30, 0.60) | 0.41 ± 0.10 |
|  |  | Aa | 7 |  |  | 1.25 | (1.06, 1.47) |  |
|  |  | AA | 5 |  |  | 1.00 | (0.83, 1.20) |  |
|  | 3 | aa | 13 | 3.46 | 0.06 | 1.30 | (1.03, 1.62) | 0.46 ± 0.06 |
|  |  | Aa | 17 |  |  | 1.24 | (0.98, 1.57) |  |
|  |  | AA | 17 |  |  | 1.00 | (0.77, 1.30) |  |
| HR16 | 1 | aa | 11 | 1.78 | 0.18 | 1.21 | (0.83, 1.76) | 0.70 ± 0.13* |
|  |  | Aa | 14 |  |  | 1.02 | (0.75, 1.40) |  |
|  |  | AA | 11 |  |  | 1.00 | (0.72, 1.39) |  |
|  | 2 | aa | 8 | 0.89 | 0.35 | 0.91 | (0.47, 1.79) | 0.37 ± 0.09* |
|  |  | Aa | 26 |  |  | 0.70 | (0.54, 0.91) |  |
|  |  | AA | 12 |  |  | 1.00 | (0.74, 1.35) |  |
|  | 3 | aa | 9 | 0.28 | 0.59 | 1.52 | (0.80, 2.86) | 0.49 ± 0.15 |
|  |  | Aa | 19 |  |  | 1.06 | (0.77, 1.47) |  |
|  |  | AA | 14 |  |  | 1.00 | (0.70, 1.44) |  |
|  | 4 | aa | 15 | 4.49 | 0.03 | 1.05 | (0.71, 1.54) | 0.59 ± 0.08* |
|  |  | Aa | 35 |  |  | 0.92 | (0.72, 1.17) |  |
|  |  | AA | 6 |  |  | 1.00 | (0.53, 1.87) |  |
| HR19 | 1 | aa | 4 | 3.47 | 0.06 | 1.48 | (0.79, 2.77) | 0.39 ± 0.10* |
|  |  | Aa | 27 |  |  | 0.87 | (0.70, 1.07) |  |
|  |  | AA | 13 |  |  | 1.00 | (0.72, 1.39) |  |
|  | 2 | aa | 10 | 0.98 | 0.32 | 1.38 | (0.96, 2.00) | 0.55 ± 0.14 |
|  |  | Aa | 23 |  |  | 1.24 | (0.94, 1.63) |  |
|  |  | AA | 7 |  |  | 1.00 | (0.68, 1.48) |  |
| HR23 | 1 | aa | 16 | 0.23 | 0.63 | 0.82 | (0.53, 1.23) | 0.52 ± 0.09 |
|  |  | Aa | 31 |  |  | 1.22 | (0.97, 1.52) |  |
|  |  | AA | 19 |  |  | 1.00 | (0.69, 1.45) |  |
|  | 2 | aa | 18 | 0.08 | 0.77 | 0.79 | (0.62, 0.99) | 0.62 ± 0.10* |
|  |  | Aa | 22 |  |  | 0.55 | (0.39, 0.78) |  |
|  |  | AA | 8 |  |  | 1.00 | (0.61, 1.64) |  |
| HR25 | 1 | aa | 13 | 0.44 | 0.51 | 0.83 | (0.64, 1.08) | 0.56 ± 0.12 |
|  |  | Aa | 24 |  |  | 1.05 | (0.85, 1.30) |  |
|  |  | AA | 16 |  |  | 1.00 | (0.75, 1.33) |  |
| G25 | 1 | aa | 22 | 0.34 | 0.56 | 0.39 | (0.29, 0.51) | 0.49 ± 0.05 |
|  |  | Aa | 44 |  |  | 0.69 | (0.60, 0.79) |  |
|  |  | AA | 17 |  |  | 1.00 | (0.87, 1.15) |  |
| G27 | 1 | aa | 17 | 1.42 | 0.23 | 0.73 | (0.53, 1.01) | 0.47 ± 0.07 |
|  |  | Aa | 34 |  |  | 0.84 | (0.68, 1.03) |  |
|  |  | AA | 9 |  |  | 1.00 | (0.54, 1.86) |  |
| G28 | 1 | aa | 10 | 3.42 | 0.06 | 0.56 | (0.27, 1.13) | 0.42 ± 0.06* |
|  |  | Aa | 36 |  |  | 0.88 | (0.72, 1.07) |  |
|  |  | AA | 12 |  |  | 1.00 | (0.78 1.29) |  |

*a* – Mean and SEM values calculated from the frequency of mutants produced by individual heterozygous coinfections; * indicate intervals that do not include 0.50.

*b –* Not available.
